# Progress in Medicine

**Published:** 1896-04-11

**Authors:** 


### Progress in Medicine.

### DISEASES OF THE NERVOUS SYSTEM.

**Neuritis.**—We have already drawn attention to Onuf's experimental observations on the degeneration of the ganglionic cells in the spinal cord following section of the motor nerves. His results would seem to indicate that the peripheral nerve lesions, such as neuritis, would be followed in many cases by similar central degenerations, and lend support to the contention of a contemporary[22] that there is nothing in the way for supposing that the different parts of a neurone, the cell and its prolongations, the nerves, may be affected separately in a pathological process, or that the nerves, the peripheral portion, are, more than the cells, the central portion, exposed to injurious influences of all sorts, such as traumatisms, intoxications, infections, &c., or that the same agent, acting upon the entire nervous unity, may determine more marked lesions in one of its constituent parts than in the other. The author refers to two cases recorded by Goldscheider and Moxter, in which myelic lesions were discovered in cases of peripheral neuritis in which there was clinically polyneuritis. Another case in point is recorded by Ballet and Dutil,[23] that of a patient who presented symptoms of polyneuritis with muscular atrophy, paresis of the upper limbs and sensory disturbances. Post-mortem, they found, in addition to the ordinary lesions of polyneuritis, morbid changes in the nerve roots and myel. In the spinal cord sections, stained by Nissl's method, the cell protoplasm was swollen and without prolongations; staining by hæmotoxylin and eosin showed the cell nucleus to be displaced and crenulate, and in certain cells stellate, while picrocarmine staining revealed nothing. Ballet stated that it is now known that whenever a nerve lesion exists, the centre with which this nerve is connected undergoes changes. In other words, let any portion of a neurone be damaged, and the lesion necessarily affects all other parts of this neurone. These views were endorsed by Marie.

Pneumonic neuritis is at any rate rare, and thus the case of neuritis of the brachial plexus as a sequel of pneumonia recorded by Lessynsky[24] is noteworthy. A man, aged 36, developed neuritis of the brachial plexus of both sides during convalescence from pneumonia, followed by the reaction of degeneration in the affected muscles.

Chipault[25] has had excellent results from stretching the plantar nerves in five cases of perforating ulcer. In two cases the ulcer was due to peripheral neuritis, and in the three others it was of spinal origin. The ulcers were cured, and the patients remained free from their affection even after several months' interval.

**Landry's Paralysis.**—Marie and Marinesco[26] record a case of Landry's paralysis with polymyelitic lesions of the nerve centres due to the presence of a microbe, which, from a morphological point of view, closely resembled the bacillus anthracis. The two most interesting points in this case, the authors believe, are: (1) On the one hand, it shows that in certain forms of Landry's paralysis comparatively well-marked central lesions exist, a fact which upsets the theories of certain authorities, who allege that this syndrome is dependent exclusively upon peripheral lesions; (2) on the other hand, it explains the pathogeny of the disease, showing that microbes apparently play the principal part in the production of the lesions. Subsequently Ballet and Dutil[27] reported a case presenting a certain analogy with that of Marie and Marinesco. Shortly after an attack of influenza, the patient, who had been an excessive drinker, developed symptoms which assumed one of the typical forms of Landry's ascending paralysis. This paralysis was possibly due to infective myelitis, though no micro-organisms were observed, but was in all probability of toxic origin. Post-mortem examination revealed the existence of generalised myelitis. The spinal cord was injected throughout, but especially in the region of the anterior cornua. This vascularisation closely resembled that seen in experimental myelitis. The cells showed evidence of early inflammatory degeneration.

[22] Med. Week, Oct. 11, 1895, [23] Med. Week, Dec. 20, 1895. [24] Jour. of Nervous and Ment. Dis., Dec., 1895. [25] Epit. Brit. Med. Journ., Oct. 19, 1895. [26] Med. Week, Oct. 25, 1895. [27] Med. Week, Nov. 1, 1895.

### FOODS AND DIGESTION.

**Tea.**—McKechnie[1] has been investigating afresh the effects of tea on digestion, and thus carrying on the work done by Sir W. Roberts. As regards peptic digestion in the test tube, if tea is added, he finds that the time of digestion is not affected by the shortness or long duration of the infusion, nor by the presence or absence of tannin. In the stomach, however, digestion was retarded and less juice was secreted when long-infused tea was used. This occurred just the same when the tannin had been removed. He concludes that tannin itself has no effect, but that some unknown substance is extracted from tea by long infusion, which hinders peptic digestion. He finds that Indian teas are freer from this substance than Chinese, contrary to the usual belief.

**Milk.**—J. L. Kerr[2] insists on the inferior value of boiled milk compared with uncooked as a food. The latter is largely composed of living cells, which are, he thinks, absorbed alive and enter the circulation without change. This view is supported by the work of Koplik,[3] who showed that the unabsorbed nitrogen in the fæces is much greater after a meal of cooked than after one of raw milk. Other writers, however, have found a more rapid increase in weight in children fed on boiled or sterilised milk than in those fed on raw. A full account of the preparation and properties of Kephir is given by C. D. Spivak[4] with the bibliography of the subject. This beverage has only been known in the scientific world for thirty years. It appears to be a preparation of cow's milk fermented by certain so-called grains, which are of the size of a walnut, and consist of masses of yeast and bacilli matted together. The resulting liquid is a food of great value in pulmonary and other wasting disorders, and possesses marked diuretic properties. Moreover, when well made it has not only a nutrient value akin to that of cod liver oil, but it has a marked effect on the digestive organs, and is often retained in the stomach when everything else is vomited. Thus Gauches and Gallois[5] recommend it in uræmic states when even milk cannot be retained, and others have used it with great success in ulcer and cancer of the stomach. Thorne Thorne lectured recently on the communication of disease by milk, pointing out that typhoid was only thus conveyed when the milk had been contaminated by a patient suffering from that disease, that scarlet fever and diphtheria might be derived on the other hand from a disease in the cow herself, and that cholera, foot and mouth disease, and tuberculosis were also carried in milk. The lecturer urged the necessity of boiling all milk in view of the great danger of infection of tuberculosis, and the difficulties of detecting the disease in the cow. The New York milk supply is described by Lederle as subject to very careful supervision, and £2,000 were imposed in fines for adulteration during the last year alone. The total solids required by law there in the milk are 12 per cent., and the fat must equal 3 per cent. These percentages, according to Dr. Lederle,[6] are too low and should be raised. The bacteria in numerous samples numbered some millions to the cubic centimetre instead of less than a hundred thousand, which was due to uncleanness in transport and storage. In spite of what had been done in destroying tubercular cattle, large numbers, probably 70 per cent., of those in New York State were tubercular. The city consumed for its daily supply the milk of 100,000 cattle. Adulteration of milk in Indian cities is discussed by J. Datta,[7] who finds it almost universal. The chief substance used for the purpose is water, often filthy and infected with cholera and other diseases. Molasses is used to make up the diminished specific gravity. Ghee and vegetable oil, which form a large part of the national diet, are also much adulterated, and the writer finds that, except for one or two local Acts, the Indian code is almost entirely inoperative in the restraint of adulteration.

**Alcoholic Drinks.**—The value of cider as a beverage is the subject of an important article in the *Lancet* of December 14th. No doubt much carelessness and unskilfulness in manufacture has damaged the market for this most wholesome drink in the past. The composition is not dissimilar to that of claret, malic acid taking the place of tartaric acid, and the alcohol not exceeding 5 per cent. Moreover, the alcohol is a remarkably pure one and free from injurious byeproducts. In France it is largely used for the manufacture of a good quality of brandy, and is rich in valuable and exhilarating ethers. The malates of cider break up into alkaline salts in the body and give to the drink its diuretic properties, which render it a useful beverage in uric acid and gouty conditions. It is, of course, only pure and moderately dry cider which is wholesome. Thus some of the Hereford varieties, for instance, do not show more than 1 per cent. of sugar, while, on the other hand, most of the manufactured and fortified samples are neither wholesome nor possessed of the refreshing properties of the pure, well-fermented article. Its use has been largely replaced by that of lager beer and light claret, " made in Germany," without any corresponding advantage to the consumer.

Chittenden and Mendel[8] have carried out further experiments on the action of alcohol upon digestion. The present tests of the chemical processes of digestion were planned with the view to eliminate the effects of increased peristalsis. Very small quantities of alcohol appeared to stimulate digestion, while larger ones inhibited it. The "fusel oils" themselves had a similar action on digestion, and the effects of whisky, rum, and brandy were not markedly different from those of pure alcohol. On the whole, the authors confirm the results of Roberts and other writers. The report of the inspector[9] under the Inebriates Acts is of interest as showing the large number of patients who are cured permanently by detention under proper care, even though twelve months is the present limit of time. More retreats and a longer period of detention are urgently needed, and the profession has shown already its unanimous approval of an alteration of the law in this direction.

**Condensed Foods.**—In the United States army an interesting trial[10] has once more been made of the possibility of saving carriage by the use of condensed foods. Compressed tablets containing the usual articles of diet in a concentrated form were supplied to a body of troops, but the men broke down utterly after four days' easy marching, and more than half were ill enough for hospital treatment, though the tabloids when moistened or boiled formed a fairly bulky food. The impossibility of feeding men on essences was long ago shown in the British army, but an emergency ration of compressed beef and vegetables is still[11] occasionally issued to our troops which takes the place of the German pea sausage. The editors of " Food and Sanitation "[12] have a fresh article on the comparative worthlessness of many of the highly recommended meat extracts and condensed food preparations. Thus they point out that the best known meat Essences and Juices contain only 2 or 3 per cent. of nutrient matter, and the ordinary Extracts of meat about 10 per cent., while much even of this meagre total is probably inactive, being a peptone derived from gelatine. Speaking of one of these much-praised meat juices they say: "As far as nutrient value goes, the water in which dinner plates are washed would be about as valuable." These statements are, if well founded, of very serious importance, since so many invalids depend in emergencies on these essences. However, the writers point out that Bovril now contains 43 per cent. of nutrient matter against the 2 or 3 per cent. found in the others.

[1] Lancet, Oct. 14. [2] Brit. Med. Jour., Dec. 14. [3] Brit. Med. Jour., Jan. 16. [4] New York Med. Jour., Jan. 18. [5] New York Med. Jour., Jan. 18. [6] Med. Rec., Jan. 18. [7] Ind. Med. Clin. Rec., Oct. [8] Amer. Jour. Med. Sc., Jan., 1896. [9] Lancet, Oct. 5. [10] Med. Rec., Nov. 30. [11] Med. Rec., Feb. 1. [12] Food and San., Dec. 14.

